# Case Report: Adrenocortical adenoma harboring atypical subclinical Cushing’s syndrome with dehydroepiandrosterone sulfate transferase and cytochrome b5 expression in tumor cells

**DOI:** 10.3389/fendo.2025.1624396

**Published:** 2025-07-23

**Authors:** Ichiro Abe, Yuto Yamazaki, Ayuko Higashi, Kentaro Ochi, Keita Kubo, Yuya Fujita, Kaori Takeshita, Toshiaki Wada, Ryo Mitsuoka, Yo Yamashita, Ryoko Yoshida, Tamotsu Kato, Tadachika Kudo, Shigero Miyajima, Tatsu Ishii, Satoshi Nimura, Takashi Suzuki, Hironobu Sasano, Kunihisa Kobayashi

**Affiliations:** ^1^ Department of Endocrinology and Diabetes Mellitus, Fukuoka University Chikushi Hospital, Chikushino, Fukuoka, Japan; ^2^ Department of Pathology, Tohoku University Graduate School of Medicine, Sendai, Miyagi, Japan; ^3^ Department of Urology, Fukuoka University Chikushi Hospital, Chikushino, Fukuoka, Japan; ^4^ Department of Pathology, Fukuoka University Chikushi Hospital, Chikushino, Fukuoka, Japan

**Keywords:** subclinical Cushing’s syndrome, hypercortisonemia, dehydroepiandrosterone, cytochrome b5, electron transfer system

## Abstract

Subclinical Cushing’s syndrome (SCS) is frequently encountered during the clinical evaluation of adrenal incidentalomas and is typically associated with reduced levels of serum dehydroepiandrosterone sulfate (DHEA-S). Cytochrome b5 is a component of the electron transfer system that enhances the activity of 17, 20-lyase relative to that of 17a-hydroxylase. Therefore, tumors harboring cytochrome b5 might be associated with dehydroepiandrosterone sulfotransferase (DHEA-ST) expression, resulting in unsuppressed serum DHEA-S levels. Here, we reported the first case of SCS with elevated serum DHEA-S levels in an incidentally detected adrenocortical adenoma showing immunohistochemical positivity for both cytochrome b5 and DHEA-ST.

## Introduction

Subclinical Cushing’s syndrome (SCS), which has been also recently reported as mild autonomous cortisol secretion (MACS), is one of the functional adrenocortical diseases, which is clinically associated with glucose intolerance, dyslipidemia, hypertension, hypokalemia, and other manifestations as commonly seen in full-blown Cushing’s syndrome (CS); however, it lacks the typical clinical features of CS (1–3). CS and SCS have recently been classified into various clinicopathological phenotypes (4–7). In general, serum dehydroepiandrosterone sulfate (DHEA-S) levels are reduced in patients with CS and SCS (8, 9). In addition, unsuppressed DHEA-S levels could be detected in the patients with adrenocortical carcinoma (10). However, unsuppressed serum DHEA-S levels have been reported in patients with CS, showing positive immunohistochemistry for dehydroepiandrosterone sulfotransferase (DHEA-ST) and cytochrome b5 (11–14). Cytochrome b5 is a component of the electron transfer system that increases the activity of 17, 20-lyase relative to that of 17a-hydroxylase (15–18). 17, 20-lyase is essential for the production of adrenal androgens, whereas 17a-hydroxylase is essential for the synthesis of cortisol (15–18). Therefore, in these patients, serum DHEA-S levels were elevated, and DHEA-ST-positive cells in patients with CS were also positive for cytochrome b5.

In the present case, the patient with SCS had both hypercortisonemia and elevated serum DHEA-S levels. Subsequent thorough histopathological analysis of the resected tumor revealed that it was immunohistochemically positive for both cytochrome b5 and DHEA-ST. However, these phenotypes have not been reported in patients with SCS. Therefore, this is the first case of SCS with increased serum DHEA-S levels, potentially due to the simultaneous expression of DHEA-ST and cytochrome b5 in tumor cells.

## Case presentation

### Clinical summary

**Figure 1:**
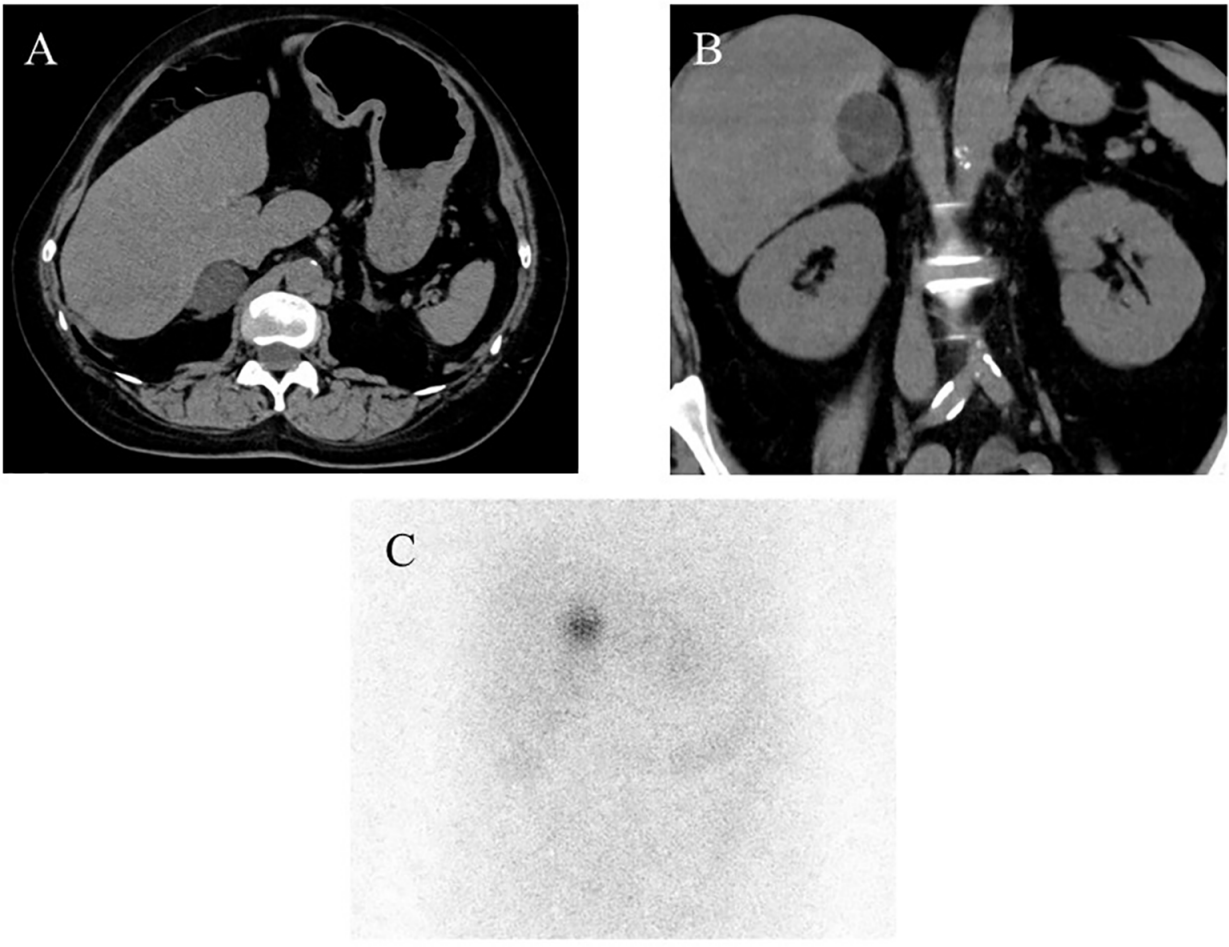
Imaging analysis. **(A, B)** Non-enhanced computed tomography (CT) demonstrated the presence of a right adrenal tumor, **(C)** 131I-adosterol adrenal scintigraphy revealed increased uptake of adosterol in the right and suppressed one in the left adrenal glands, respectively.

A 57-year-old Japanese woman was referred to Fukuoka University Chikushi Hospital and admitted for the management of glucose intolerance and assessment of diabetic complications. Her body mass index was 24.2 kg/m^2^, and she did not have any clinical symptoms suggestive of CS. She was treated with nifedipine (40 mg/day) and bisoprolol (2.5 mg/day) for hypertension; pitavastatin (1mg/day) for dyslipidemia; as well as insulin (35 U/day), sitagliptin (50 mg/day), and ipragliflozin (50 mg/day) for diabetes mellitus. During hospitalization, a nodular lesion was incidentally detected in her right adrenal gland (31 mm in diameter) on non-enhanced abdominal computed tomography (CT) (**Figures 1A, B**). Subsequently, clinical and endocrinological examinations were performed (**Table 1**). Her morning plasma adrenocorticotropic hormone (ACTH) level was low (<1.5 pg/mL), whereas her serum cortisol level was within normal limits (17.5 µg/dL). Besides, serum cortisol levels were not suppressed by the 1-mg dexamethasone suppression test (17.8 µg/dL), and the nocturnal serum cortisol levels were elevated (18.1 µg/dL). These results met the diagnostic criteria for SCS (9, 19). Her plasma renin activity was 7.8 ng/mL/hr, and plasma aldosterone concentration was 14.2 pg/mL. The ratio of plasma aldosterone concentration to plasma renin activity (ARR) was 1.84. The plasma/urinary catecholamine and urinary metanephrines values were within normal limits. Notably, serum DHEA-S levels were unsuppressed (141 µg/dL (normal range: 8-188 µg/dL)) despite the suppressed hypothalamic-pituitary-adrenal (HPA) axis (DHEA-S levels were measured using Chemiluminescent Enzyme Immunoassay (SRL, Inc., Japan)). ^131^I-adosterol adrenal scintigraphy revealed increased uptake of adosterol by the tumor in the right adrenal gland, and suppressed uptake in the left adrenal gland (**Figure 1C**). Subsequently, a laparoscopic resection of the right adrenal tumor was performed. After surgery, she was placed on hydrocortisone replacement therapy. The serum cortisol levels remained low (0.2 µg/dL) at 7 days after the operation, whereas the serum DHEA-S levels decreased (20 µg/dL). Regarding the clinical findings, glucose intolerance improved, and the medication dosage was decreased (insulin, 19 U/day; sitagliptin, 50 mg/day). In addition, hypertension improved, which led to a decrease in nifedipine dosage (10 mg/day). Moreover, pitavastatin was discontinued.

**Table 1:** Preoperative characteristics of the patient.

	Values or details
Height (cm)	153.6
Body weight (%)	57.0
BMI (kg/m^2^)	24.2
Systolic Blood Pressure (mmHg)	132
Diastolic Blood Pressure (mmHg)	90
Medication for hypertension	nifedipine: 40 mg/day, bisoprolol: 2.5 mg/day
FPG (mg/dL)	121
HbA_1c_ (%)	6.4
Medication for diabetes mellitus	insulin: 35 U/day, sitagliptin: 50mg/day, ipragliflozin: 50 mg/day
LDL-C (mg/dL)	148
HDL-C (mg/dL)	46
TG (mg/dL)	119
Medication for dyslipidemia	pitavastatin: 1mg/day
ACTH (pg/mL)	< 1.5
Cortisol (µg/dL)	17.5
Renin activity (ng/mL/hr)	7.8
Aldosterone (pg/mL)	14.2
Adrenaline (pg/mL)	11
Noradrenaline (pg/mL) (pg/mL)	231
Dopamine (pg/mL)	7
DHEA-S (µg/dL)	141
Nocturnal cortisol at 21:00 (µg/dL)	18.1
Cortisol after a 1-mg DST (µg/dL)	17.8

BMI, body mass index; FPG, fasting plasma glucose; HbA_1c_, glycated hemoglobin; LDL-C, low-density lipoprotein cholesterol; HDL-C. high-density lipoprotein cholesterol; TG, triglyceride; ACTH, adrenocorticotropic hormone; DHEA-S, dehydroepiandrosterone sulfate; DST, dexamethasone suppression test.

### Pathological findings

**Figure 2:**
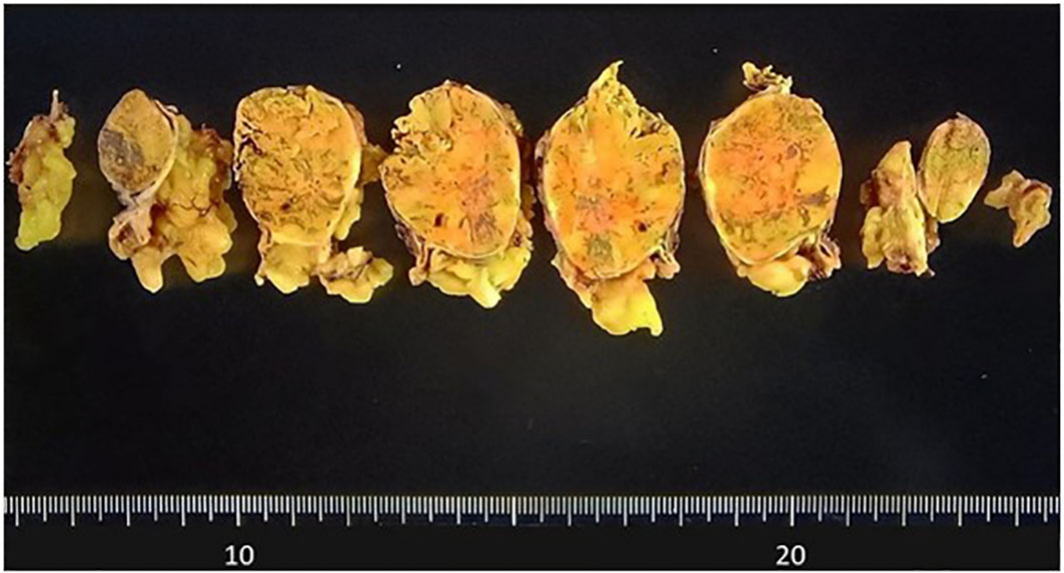
Macroscopic findings of the resected specimen. The well-circumscribed tumor measuring 31mm in greatest dimension appeared yellow on the cut surface.

**Figure 3:**
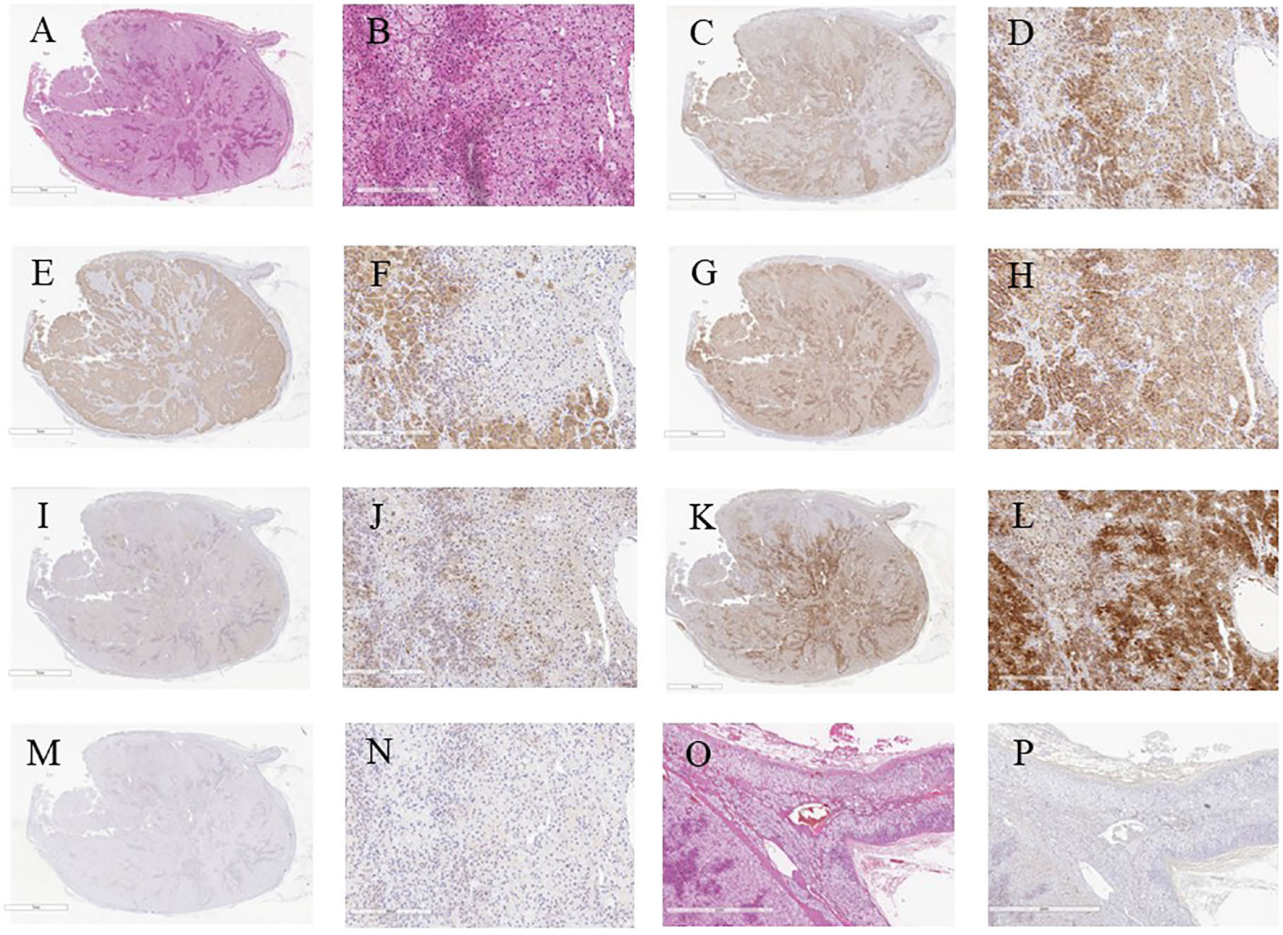
Microscopic and immunohistochemical findings of the resected specimen. **(A)** Low power view of the whole cut section (Hematoxylin and eosin-staining). **(B)** Hematoxylin and eosin-stained tumor section on high magnification showing large polygonal tumor cells with abundant clear foamy cytoplasm (X40). **(C-N)** Serial tissue sections immunostained with steroidogenic enzymes: **(C, D)** HSD3B, **(E, F)** CYP17A, **(G, H)** CYP11B1, **(I, J)** DHEA-ST, **(K, L)** Cytochrome b5, **(M, N)** CYP11B2. **(O, P)** Serial sections of adjacent non-neoplastic adrenal gland: **(O)** Hematoxylin and eosin-stained, **(P)** DHEA-ST immunohistochemistry. HSD3B, 3β-hydroxysteroid dehydrogenase; CYP17A, cytochrome P450 family 17 subfamily A; CYP11B1, cytochrome P450 family 11 subfamily B member 1; DHEA-ST, dehydroepiandrosterone sulfotransferase; CYP11B2, cytochrome P450 family 11 subfamily B member 2.

**Figure 4:**
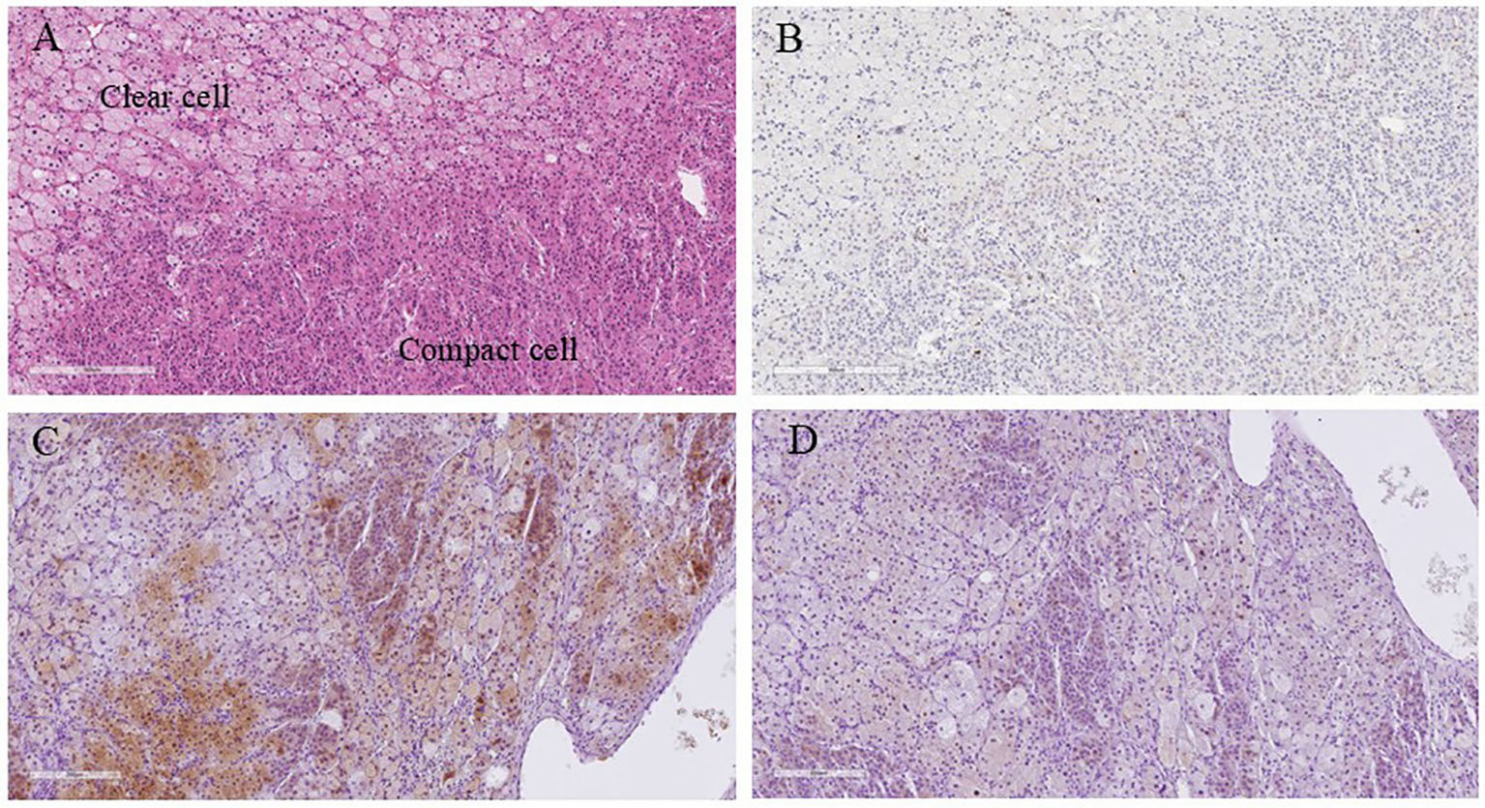
Higher magnification microscopic images of the resected specimen. **(A)** Hematoxylin and eosin-stained tumor section (X100); **(B)** Ki-67 immunohistochemistry (X100); **(C)** Cytochrome b5 immunohistochemistry (X100); **(D)** DHEA-ST immunohistochemistry (X100). The co-expression of cytochrome B5 and DHEA-ST was investigated using mirror image analysis. DHEA-ST, dehydroepiandrosterone sulfotransferase.

Macroscopically, a well-circumscribed yellowish lesion of the adrenal gland was detected in the resected specimen (**Figure 2**). Microscopically, the adrenocortical lesion was composed of large polygonal tumor cells with abundant clear foamy cytoplasm and partly composed of compact cells. In addition, small polygonal clear cells and small compact cells were also intermingled with the large cells above. The clear cells were immunohistochemically positive for steroidogenic enzymes such as 3β-hydroxysteroid dehydrogenase (3BHSD), cytochrome P450 family 17 subfamily A (CYP17A), and cytochrome P450 family 11 subfamily B member 1 (CYP11B1), whereas the small compact cells were negative for 3BHSD but positive for both CYP17A and CYP11B1. In addition, the compact cells were both positive for DHEA-ST and cytochrome b5. Cytochrome P450 family 11 subfamily B member 2 (CYP11B2)-positive cells were not detected in the tumor (**Figures 3A–N**, **4A–D**). The Ki-67 labeling index of the tumor was less than 2% in the tumor, The criteria of Weiss (1/9: only clear cells in the cytoplasm were detected) revealed that the tumor was histopathologically diagnosed as an adrenocortical adenoma. No significant differences of Ki-67 labeling index and nuclear morphology were detected between clear and compact tumor cells (**Figures 4A, B**). In adjacent adrenal gland, marked atrophic changes of zona fasciculata and zona reticularis within adjacent adrenal cortex were detected. Paradoxical hyperplasia of the zona glomerulosa was not detected in the adjacent adrenal gland. DHEA-ST immunoreactivity of zona reticularis within adjacent adrenal cortex was clearly diminished, which histologically reflected the suppressed status of HPA axis in a long-term period consistent with the presence of autonomous cortisol overproduction (**Figures 3O, P**).

## Discussion and conclusion

This patient was clinically diagnosed with atypical SCS. Our previous study of 61 cases of adrenal incidentalomas revealed that 10 (16.4%) patients had SCS. and they all showed improved clinical and hormonal findings following adrenalectomy (19). In the present case, although the patient was considered to have atypical SCS, surgical resection also improved the complications of all metabolic disorders. Glucose intolerance, hypertension, and dyslipidemia improved after tumor extirpation.

Endocrinological investigations have also demonstrated clinical remission of SCS following adrenalectomy. These findings highlighted the clinical importance of an accurate diagnosis of adrenal incidentalomas. Histopathological analysis demonstrated that the tumor produced cortisol, similar to other cases of SCS. However, in contrast to patients with typical SCS, this particular patient exhibited elevated serum DHEA-S levels before surgery, and immunohistochemistry revealed DHEA-ST-positive cells in the tumor. The criteria of Weiss revealed that the tumor was histologically diagnosed as an adrenocortical adenoma. Decreased serum DHEA-S levels after tumor extirpation were consistent with these findings. In addition, of particular interest, cytochrome b5 was detected in the DHEA-ST-positive cells. Biochemically, the electron transfer system could modulate the relative activity of 17, 20-lyase to 17a-hydroxylase, and cytochrome b5 is an important component of the electron transfer system that increases the relative activity of 17, 20-lyase, which is essential for the production of adrenal androgens, relative to that of 17a-hydroxylase, which is a prerequisite for cortisol biosynthesis (15–18). Therefore, the expression of cytochrome b5 could have contributed to the elevated serum DHEA-S levels and increased DHEA-ST-positive cells in the present case. In addition, both DHEA-ST and cytochrome b5 were positive for CYP17A and CYP11B1 but negative for 3BHSD in tumor cells of this patient. These results demonstrated the increased relative activity of 17, 20-lyase relatively to that of 17a-hydroxylase led to elevated serum DHEA-S levels. Besides, cortisol production in both cytochrome b5 and DHEA-ST positive cells decreased as a result of 3BHSD negativity in tumor cells. Therefore, these histopathological findings were consistent with the unusually high preoperative serum DHEA-S levels observed in the present patient. The patient with an adrenocortical adenoma associated with elevated glucocorticoids and 11-oxygenated androgens but not elevated DHEA-S levels was reported to be positive for cytochrome B5 immunoreactivity (20). Therefore, the efficacy and function of cytochrome B5 could result in various phenotypes. In addition, the analysis of setum DHEA-S levels in 38 patients with SCS demonstrated that some patients with SCS had unsuppressed DHEA-S levels. Besides, not elevated baseline serum cortisol levels, but unsuppressed serum cortisol levels in the 1-mg dexamethasone suppression test, were proposed to be associated with low serum DHEA-S levels (8). However, the present patient had unsuppressed serum cortisol levels in the 1-mg dexamethasone suppression test and unsuppressed serum DHEA-S levels. Considering those above, further cases should be required in order to reveal the detailed mechanisms of atypical SCS.

In summary, we report the first case of SCS with elevated serum DHEA-S levels due to the simultaneous expression of both DHEA-ST and cytochrome b5 in tumor cells. The results of this study emphasize the importance of detailed histopathological evaluation of incidentally detected resected adrenal specimens. In addition, the mutation status of the patient was not evaluated in this case. Therefore, further investigations such as the analysis of a large cohort and/or more detailed investigations of the tumor, such as genomic evaluations, are warranted.

## Data Availability

The original contributions presented in the study are included in the article/supplementary material. Further inquiries can be directed to the corresponding author.
